# CD28 expression in sentinel node biopsies from breast cancer patients in comparison with CD3-ζ chain expression

**DOI:** 10.1186/1479-5876-2-45

**Published:** 2004-12-21

**Authors:** Jana M Schüle, Leif Bergkvist, Leif Håkansson, Bertil Gustafsson, Annika Håkansson

**Affiliations:** 1 Department of Surgery, Central Hospital, SE-72189 Västerås, Sweden; 2 Center for Clinical Research of Uppsala University, SE-72189 Västerås, Sweden; 3 Department of Oncology, University Hospital of Linköping, SE-58185 Linköping, Sweden; 4 Department of Pathology and Cytology, University Hospital of Linköping, SE-58185 Linköping, Sweden

## Abstract

**Background:**

Immunosuppression is documented in several malignant diseases, including breast cancer. Subsequently, future therapeutic concepts might include immunological approaches. However, detailed knowledge about tumor immunogenicity and host immunoreactivity, and how to assess these adequately, is still limited. We studied CD28 and CD3-ζ expression in sentinel node biopsies (SNB) from breast cancer patients to analyze tumor-related changes in T cell activity.

**Method:**

25 women underwent surgery for primary breast cancer, including SNB. Frozen sections from 21 sentinel nodes could be analyzed with a double-staining technique. CD28 expression was studied in CD4+ and CD8+ T-lymphocyte subsets and compared with CD3-ζ expression in three specified nodal regions.

**Results:**

The degree of CD28 expression varied between the different lymph node areas. The lowest degree of CD28 expression was observed in CD4+ T-lymphocytes in the paracortex and germinal centers. Here, a good agreement with CD3-ζ expression was found. A higher CD28 expression was noted in CD4+ T-cells in the primary follicles, where concordance with CD3-ζ expression was weaker. The CD8+ T-lymphocyte subset displayed generally a higher degree of CD28 expression than the CD4+ subset.

**Conclusion:**

Sentinel lymph nodes from breast cancer patients displayed local immunosuppression of varying extent. In the areas with the lowest degree of CD28 expression an accordingly low CD3-ζ expression was found. The SNB might prove an important diagnostic tool for the evaluation of interactions between tumor and the host immune system, helping to select patients who might benefit from adjuvant immunotherapy.

## Background

Numerous studies [1] portray a decreased anti-tumor immunoreactivity in patients with malignancies, including breast cancer [2-4], and its correlation with disease progression and survival [5,6]. Antigen presentation and subsequent T-cell activation play a major role in initiating and maintaining an adequate anti-tumor response. However, the complex signaling cascades engaged in this process are not yet fully understood, rendering it difficult to be successfully addressed in therapeutical approaches. Better knowledge of these mechanisms is therefore essential for further development of immunological treatment strategies.

The CD28 surface receptor is normally expressed on 95% of CD4+ T-cells and approximately 50% of CD8+ T-cells in human peripheral blood [7]. Its natural ligands, the B7 molecules, are found on various antigen-presenting cells [8]. CD28 expression increases in activated T-cells [9]. Ligation of CD28 possesses major importance as a second, co-stimulatory signal during antigen/MHC complex presentation [10], hereby leading to a lower T-cell activation threshold and a longer duration of the proliferative response [11]. However, activation via the T-cell receptor alone induces transient T-cell proliferation [12], T-cell anergy, or deletion [13].

Decreased CD28 expression is described in dysfunctional peripheral T-lymphocytes from patients with hairy cell leukemia [14] and chronic lymphocytic leukemia [15]. In colorectal cancer, tumor-infiltrating lymphocytes (TIL) lack CD28 in contrast to those in normal colon interstitium [16]. This is consistent with findings in TIL from primary melanoma patients [17]. In melanoma metastases, CD28 down-regulation is more pronounced in areas of tumor regression [18,19]. Compared to healthy controls, breast cancer patients display significantly lower percentages of CD28+ T cells in peripheral blood [20]. To our knowledge, no studies as to the expression of CD28 in sentinel node biopsies from breast cancer patients have yet been published.

The expression of the zeta chain of the T-cell receptor (CD3-ζ) is decreased in sentinel node biopsies from breast cancer patients [21]. This down-regulation is most pronounced in the paracortex, the main T-cell activation area. In the present study, CD28 expression was analyzed in the same material and subsequently compared with CD3-ζ expression in parallel sections.

## Methods

### Study population

The study comprised 25 patients who underwent surgery for primary breast cancer, using the sentinel node biopsy technique. Inclusion criteria for enrolment in the study protocol were informed patient consent and a newly diagnosed palpable invasive breast cancer. Exclusion criteria were palpable axillary metastases, multifocality of the cancer, ongoing pregnancy or preoperative cytotoxic treatment.

In two cases, the sentinel node could not be immunologically analyzed due to lack of technical quality. In two other cases, nodal tumor growth was too abundant and remaining lymphoid tissue too little to analyze the sections. Thus, the remaining study population comprised 21 patients. Patient and tumor characteristics are presented in Table 1.

**Table 1:** Tumor and other selected characteristics of 21 women operated on for primary breast cancer.

	Median (range)	n (%)
Age	60 (36–86)	
***Menopausal status***		
premenopausal		5 (24)
postmenopausal		16 (76)
***Nodal status***		
negative SNB		12 (57)
positive SNB		9 (43)
only positive SNB		7 (33)
3–4 positive lymph nodes		2 (10)
***Tumor stage***		
I		7 (33)
IIa		7 (33)
IIb		7 (33)
***Histological type***		
ductal invasive		17 (81)
lobular		3 (14)
mucinous		1 (5)
Tumor size (mm)	21 (1–60)	
***Estrogen receptor status***		
positive		19 (90)
negative		2 (10)
***DNA ploidy status***		
euploid		9 (43)
aneuploid		12 (57)
***S phase***		
high		3 (14)
low		18 (86)
***(Elston) histological grade***		
I		2 (10)
II		11 (52)
III		7 (33)
Not measured		1 (5)

SNB = sentinel node biopsy

The study protocol was approved by the ethics committees at the University of Uppsala and the University Hospital of Linköping.

### Identification of sentinel node

Sentinel nodes were identified by injection of a radioactive tracer (Tc-99-nanocolloid) close to the tumor site, preoperative lymphoscintigrams, and injection of Patent blue dye (Guerbet, Paris, France). After biopsy, the sentinel nodes were sent fresh to the pathology department. Specimens were snap-frozen and tissue sections (6–7 μm thick) were obtained for immediate diagnostic analysis regarding the occurrence of metastasis in the sentinel node. Additional frozen sections were wrapped in parafilm and stored at -70°C until processed further at University Hospital of Linköping. Routine diagnostic studies of both tumor tissue and lymph nodes were performed at the pathology department of Central Hospital, Västerås and comprised all parameters listed in Table 1.

### Monoclonal antibodies

The monoclonal antibodies used were as follows: CD4 (Clone SK3, Becton-Dickinson, Stockholm, Sweden), CD8 (Clone SK1, Becton-Dickinson, Stockholm, Sweden), CD28 (Clone L293, Becton-Dickinson, Stockholm, Sweden), CD3 (Clone UCHT1, DAKO Stockholm, Sweden), TCR-zeta (Clone 2H2D9 (TIA-2), Immunotech, Stockholm, Sweden).

### Preparation of node biopsies and immunological staining of tissue sections

Tissue sections obtained as described above were fixed with 4% paraformaldehyde (pH 7.4) (Riedel-de Haen AG, Seelze, Germany), supplemented with 5.4 g/L of glucose for 5 min and soon afterward washed three times in Hanks' balanced salt solution (BSS; Gibco, Paisley, United Kingdom) supplemented with 0.01 M HEPES solution. To avoid unspecific binding, sections were blocked with normal rabbit serum before the first staining and subsequently incubated with the primary antibodies CD3 (1/40), CD4 (1/25) and CD8 (1/50) for 30 minutes. After the slides had been washed in BSS/saponin, biotinylated rabbit anti-mouse immunoglobulin was added at a 1/100 dilution in BSS/saponin. Mouse IgG (Sigma, Stockholm, Sweden) was used as a negative control. The slides were then incubated with peroxidase-labeled streptavidine (P0397; Dako, Stockholm, Sweden) at a 1/100 dilution in BSS/saponin for 30 minutes. DAB (3,3'-diaminobenzidine, D-5637, Sigma, Stockholm, Sweden) was used as a substrate, which resulted in a brown color. The expression of the zeta chain or CD28 was identified using mouse monoclonal antibodies to these substances. The sections were first blocked by incubation with normal goat serum and subsequently incubated with the primary antibodies. Sections stained for the zeta chain (1/2.5) were incubated for 30 minutes while sections stained for CD28 (1/25) were incubated over night. The slides were again washed in BSS/saponin and incubated with goat anti-mouse immunoglobulin for 30 minutes and then with the alkaline phosphatase-anti-alkaline phosphatase (APAAP) mouse monoclonal antibody (Dakopatts D 651) at a dilution of 1/25 in BSS/saponin. After washes in BSS/saponin and Tris-buffered saline (TBS) and incubation with the alkaline phosphatase substrate (Naphthol AS-MX), 2 mg phosphate (Sigma N4875), 0.2 ml dimethylformamide, 9.8 ml 0.1 M Tris buffer pH 8.2, 50 μl 1 M levamisole (Sigma L-9756) and 10 mg Fast-Red TR salt (Sigma F 1500) for 20 minutes, the sections were again washed in TBS. Thereafter, the sections were counterstained in Mayer's haematoxylin for 15 minutes and mounted in Glycergel (Dakopatts, Sweden). All antibody solutions contained 2% normal blood donor AB serum and all incubations were performed in a moist chamber. The APAAP technique resulted in bright red staining for CD3-ζ and CD28. Double-stained cells appeared as red-brown; those with down-regulated CD3-ζ or CD28 were dominated by a brown color.

### Evaluation of occurrence and distribution of CD28

Two independent investigators (A.H., B.G.) analyzed whole sections of the sentinel nodes. The degree of CD28 expression was evaluated in both CD4+ and CD8+ T-cell subsets within three lymph node areas: primary follicles, secondary follicles (germinal centers) and paracortex. The number of CD4+ and CD8+ T-lymphocytes expressing CD28 was semiquantitatively scored as "high" (>75% CD28+), "moderate" (50–75% CD28+) and "low" (<50% CD28+). The regional scores were then put into correlation with our earlier data on expression of CD3-ζ in parallel sections of the same patients where the same scoring system was applied [21]. There was good agreement between the scores of the two investigators (80%). The few discrepancies between scores were discussed and the reasons for the difference in the scores could be identified. Thus, a consensus regarding the proper evaluation could always be reached.

### Statistical analysis

For testing correlations between the patient data depicted in Table 1 and expression of CD28 and CD3-ζ, the Mann-Whitney U test was used. The Kruskal-Wallis test was applied for comparing the three analyzed lymph node regions regarding the expression of the respective markers and for the comparison of CD28 expression in the T-cell subgroups (CD4+ and CD8+) within the same lymph node region. A p-value less than 0.05 was considered significant. For testing the agreement between the expression of CD28 (in the two separate T-cell subsets) and the expression of CD3-ζ for every nodal area separately, the Prevalence-Adjusted Bias-Adjusted Kappa (PABAK) was used. It has the same interpretation as Cohen's Kappa [22]. A value of >0.80 indicates excellent agreement, 0.61–0.80 good agreement, 0.41–0.60 moderate agreement, 0.21–0.40 fair and 0.00–0.20 poor agreement. Values less than zero suggest that the agreement is worse than expected by chance.

## Results

### Expression of CD28

The degree of CD28 expression varied considerably, both between individual patients and between different regions of the sentinel lymph nodes. The lowest degree of CD28 expression was seen in CD4+ T-lymphocytes located in the paracortex and the secondary follicles. CD8+ T-lymphocytes displayed a significantly higher degree of CD28 expression than CD4+ T-cells in both paracortex (p < 0.001) and primary follicles (p < 0.001). There were statistically significant differences in CD28 expression in CD4+ T-cells comparing the three nodal regions (p < 0.01), with CD28 expression being higher in primary follicles than in secondary follicles and paracortex. No such differences were found analyzing CD28 expression in CD8+ T-lymphocytes. No significant correlation could be found between the degree of CD28 expression and tumor size, histological type, hormone receptor status, DNA ploidy status, Elston histological grade, S-phase, patient age, nodal status or tumor stage following the TNM classification of malignant tumors, neither were there any significant correlation detected regarding clinical data and the degree of CD3-ζ expression in our previously published material [21].

### Primary follicles

**Figure 1:**
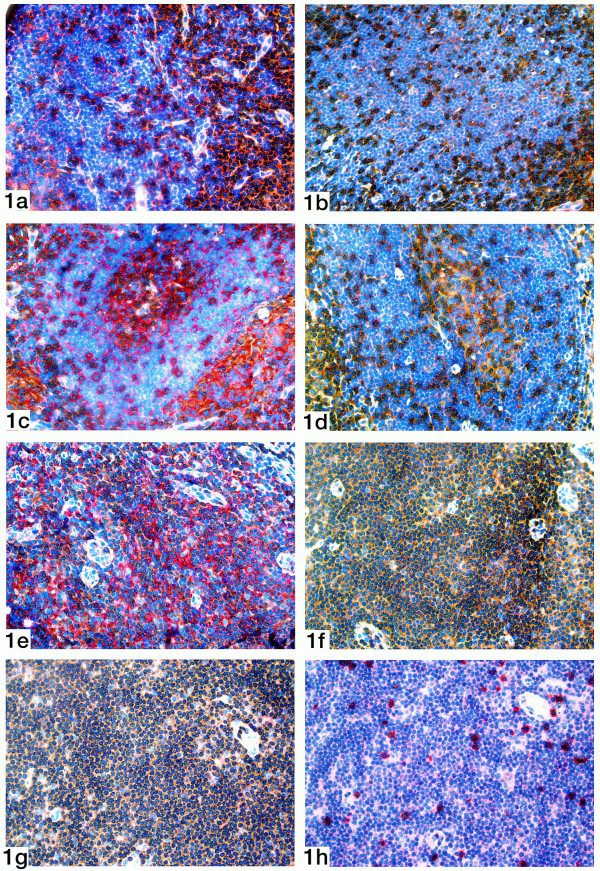
Double staining of a sentinel node for CD4 (brown staining) and CD28 (red staining).**1a). **Primary follicle showing a high expression of CD28.**1b). **Primary follicle showing a low expression of CD28. **1c). **Germinal center showing a moderate expression of CD28. **1d). **Germinal center showing a low expression of CD28. **1e). **Paracortical area showing a moderate expression of CD28. **1f). **Paracortical area showing a low expression of CD28. **1g).** Double staining of a sentinel node for CD3 (brown  staining) and the zeta chain (red staining).  Paracortical area showing a low expression of the zeta  chain. **1h).** Double staining of a sentinel node for CD8 (brown  staining) and CD28 (red staining). Corresponding  paracortical area showing a high expression of CD28.

Primary follicles are cortical lymph node areas housing predominantly B-lymphocytes. The analysis could be performed in 20 cases. In the CD4+ subset (Table 2), 7 of 20 patients displayed a high degree of CD28 expression (Figure 1a), 5 of 20 a moderate and 8 of 20 a low degree of CD28 expression (Figure 1b).

**Table 2:** Degree of CD28 expression in the CD4+ T-lymphocyte subset in different areas of the sentinel nodes from 21 breast cancer patients (numbers are frequencies).

	**Expression of CD28 on CD4+**		
	
	**High**	**Moderate**	**Low**
**Primary follicles**	7	5	8
**Secondary follicles**	1	3	12
**Paracortex**	0	5	15

In the CD8+ subset of T-lymphocytes (Table 3), a high degree of CD28 expression was found in all cases except two, which displayed a moderate degree.

**Table 3:** Degree of CD28 expression in the CD8+ T-lymphocyte subset in different areas of the sentinel nodes from 21 breast cancer patients (numbers are frequencies).

	**Expression of CD28 on CD8+**		
	
	**High**	**Moderate**	**Low**
**Primary follicles**	19	2	0
**Paracortex**	15	5	1

### Germinal centers

These B-cell areas, which develop as secondary follicles upon antigenic stimulation, could be analyzed in 16 patients. In the remaining five cases, no or very few germinal centers were found. In the CD4+ subset (Table 2), 4 of 16 patients exhibited a high or moderate degree of CD28 expression (Figure 1c), and 12 of 16 a low degree (Figure 1d). The small numbers of CD8+ T-lymphocytes in this compartment did not permit immunological analysis in any of the cases.

### Paracortex

A markedly low expression of CD28 was observed in the paracortex, the principal T-cell activation area. Sections of 20 sentinel nodes could be analyzed. In the CD4+ subset (Table 2), none of the cases established a high CD28 expression, while it was moderate in 5 of 20 patients (Figure 1e), and low in 15 (Figure 1f). In contrast, 15 of 21 cases displayed a high CD28 expression in the CD8+ subset, 5 of 21 cases a moderate, and only one case a low CD28 expression (Table 3).

### Comparison between CD28 and CD3-ζ expression

As described in a previous paper of our group [21], a low expression of CD3-ζ was seen in the primary follicles in 6 of 24 patients, in the germinal centers in 13 of 18 patients and in the paracortex in 19 of 24 patients. In the present study, the expression of CD28 was analyzed in parallel sections from the same patients.

The expression of CD28 and CD3-ζ was always compared in the same area of the same lymph node. Degrees of expression were only considered corresponding if a patient displayed exactly the same degree of expression (i.e. low, moderate or high) of both markers in that area.

In primary follicles, corresponding degrees of expression were found in 12 of 20 patients analyzing the CD4+ subset (Table 4), with a PABAK value of 0.40. Nevertheless, some sentinel node biopsies with a high CD3-ζ expression indicated a low (n = 3) or moderate (n = 3) CD28 expression on CD4+ T-cells. This discrepancy is hardly due to the presence of CD8+ T-cells as these cells presented a low or moderate CD28 expression in only one of these six biopsies. In the CD8+ subset, only 10 of 21 patients showed corresponding degrees (Table 7), resulting in a PABAK value of 0.21.

**Table 4:** Degree of CD28 expression on **CD4+ **T-lymphocytes in comparison with CD3-ζ expression in **primary follicles**. The table comprises 20 cases, where both analyses could be done in the same sentinel node biopsies (numbers are frequencies).

**Expression of CD3-ζ**	**Expression of CD28 on CD4+**		
	
	**High**	**Moderate**	**Low**
**High**	5	3	3
**Moderate**	1	2	0
**Low**	1	0	5

**Table 7:** Degree of CD28 expression on **CD8+ **T-lymphocytes in comparison with CD3-ζ expression in the **primary follicles**. Parallel sections of all 21 sentinel node biopsies were analyzed (numbers are frequencies).

**Expression of CD3-ζ**	**Expression of CD28 on CD8+**		
	
	**High**	**Moderate**	**Low**
**High**	10	1	0
**Moderate**	4	0	0
**Low**	5	1	0

In secondary follicles, 11 of 15 patients had the same degrees of CD28 and CD3-ζ in the CD4+ subset (Table 5), corresponding a PABAK value of 0.60. As earlier mentioned, the CD8+ subset was excluded from immunological analysis in this area due to the small numbers of CD8+ T-cells present.

**Table 5:** Degree of CD28 expression on **CD4+ **T-lymphocytes in comparison with CD3-ζ expression in **secondary follicles **(germinal centers). The table comprises 15 cases, where both analyses could be done in the same sentinel node biopsies (numbers are frequencies).

**Expression of CD3-ζ**	**Expression of CD28 on CD4+**		
	
	**High**	**Moderate**	**Low**
**High**	0	1	1
**Moderate**	0	1	1
**Low**	0	1	10

In the paracortex, the degree of CD28 and CD3-ζ expression was corresponding in 15 of 20 cases in the CD4+ subset (Table 6). Here, the highest PABAK was observed, reaching a value of 0.62. As the vast majority of T-lymphocytes in the paracortex belonged to the CD4+ subset, the expression of the zeta chain by CD3+ T-cells actually represented the expression by CD4+ lymphocytes. In the CD8+ subset, only 2 of 21 patients displayed corresponding degrees (Table 8), leading to a PABAK value of -0.36. Instead, 11 patients with a low degree of CD3-ζ expression (figure 1g) showed a high CD28 expression in parallel sections (figure 1h).

**Table 6:** Degree of CD28 expression on **CD4+ **T-lymphocytes in comparison with CD3-ζ expression in the **paracortex**. The table comprises 20 cases, where both analyses could be done in the same sentinel node biopsies (numbers are frequencies).

**Expression of CD3-ζ**	**Expression of CD28 on CD4+**		
	
	**High**	**Moderate**	**Low**
**High**	0	0	1
**Moderate**	0	2	1
**Low**	0	3	13

**Table 8:** Degree of CD28 expression on **CD8+ **T-lymphocytes in comparison with CD3-ζ expression in the **paracortex**. Parallel sections of all 21 sentinel node biopsies were analyzed (numbers are frequencies).

**Expression of CD3-ζ**	**Expression of CD28 on CD8+**		
	
	**High**	**Moderate**	**Low**
**High**	1	0	0
**Moderate**	3	0	0
**Low**	11	5	1

## Discussion

CD28 expression was studied in CD4+ and CD8+ T-lymphocyte subsets and compared with CD3-ζ expression in sentinel node biopsies from breast cancer patients. The lowest expression of CD28 was found in the CD4+ subset in the paracortex and germinal centers. In these areas, a moderate to good agreement between expression of CD28 on CD4+ T-cells and expression of CD3-ζ was observed. In the primary follicles, where CD28 expression on CD4+ T-cells was higher, the concordance between the two markers was less pronounced.

The CD8+ subset of T-lymphocytes displayed generally a higher degree of CD28 expression than the CD4+ subset. This applied for all analyzed regions of the lymph nodes. Moreover, the congruence with CD3-ζ expression was weaker than in the CD4+ subset.

The different functions of CD4+ and CD8+ T lymphocyte subsets are well documented. Some investigators suggest that CD8+ T-lymphocytes be closer connected to the CD28/B7 pathway than CD4+ T-cells [23]. Moreover, higher levels of B7 seem to be necessary to evoke an activation of CD8+ T-cells equivalent to CD4+ T-cells [24]. CD8+ T-cells from HIV positive individuals illustrate a more pronounced CD28 down-regulation than CD4+ T-cells, and their weakened proliferative response is marked by decreased Ca influx [25]. The presented data show a higher CD28 expression in CD8+ than in CD4+ T-cells, which stands in contrast to findings described in peripheral blood from healthy donors [7]. Thus, our results might suggest altered functions of CD4+ and CD8+ T-lymphocytes in the context of anti-tumor immunoreactivity.

It was proposed that T-lymphocytes from secondary lymphoid organs, rather than from peripheral blood, should be used to assess host immunoreactivity [26]. In bone marrow-derived T-cells from breast cancer patients, immunological changes are more pronounced than in peripheral blood lymphocytes [27,28]. Several studies document decreased CD28 and CD3-ζ expression in peripheral blood from breast cancer patients [2,4,20] in comparison to healthy individuals. However, there are no immunological data from axillary sentinel lymph nodes from normal controls. Even if such data would be most valuable in the interpretation of the present findings, it seems ethically unjustifiable to obtain such material. Still, the sentinel node biopsy might be the method of choice for assessing early immunological alterations in that the host immune response against tumors is initiated here. To confirm its distinct key position, future studies should include the comparative analysis of non-sentinel axillary lymph nodes in a distance-dependent fashion. In the present study, the down-regulation of CD28 and CD3-ζ clearly seems to be more accentuated in sentinel node biopsies than otherwise described in breast cancer patients.

Induction of T-cell anergy is one of the major tumor escape mechanisms [1], and its abrogation is therefore a main focus in the development of immunological anti-cancer strategies. Co-stimulation via the CD28/B7 pathway can prevent the induction of anergy in T-cell clones [29], but it is assumed that this might not be sufficient to reverse T-cell anergy once it is established [30]. Addition of exogenous IL-2 promotes reversal of T-cell anergy in this situation [31]. It was even suggested that decreased CD28 expression might be the result of continuous antigenic stimulation, aiming at reconstituting the non-responsiveness of T-cells [32]. The high numbers of CD4+/CD28- T-cells found in patients with rheumatoid arthritis are consistent with this theory [33]. Thus, the functional significance of decreased CD28 expression remains complex to interpret.

The functions of CD28 and CD3-ζ might be closely linked together. Engineered T-cells co-delivering CD28 activation in addition to the T-cell receptor (TCR) subunit CD3-ζ are more effective to activate an anti-tumor response in vivo than T-cells with TCR-CD3-ζ only [34]. Without co-stimulatory signaling via CD28/B7, tyrosine phosphorylation of the zeta chain of the T-cell receptor is inhibited [35]. Moreover, signaling via CD3-ζ could activate protein-tyrosine kinases that subsequently augment signal transduction via CD28/B7 [36]. The present data document a good agreement in the expression of the two markers in areas of pronounced down-regulation.

Several studies point towards a role for immunological treatment approaches in breast cancer [37-39]. The present data on decreased expression of CD28 and CD3-ζ in sentinel lymph nodes confirm that breast cancer has an impact on local immunoreactivity. However, the occurrence of varying individual patterns could be explained by the existence of both non-immunogenic and immunogenic, if not immunotoxic tumor types. Tumors with little inherent immunogenicity might not be as susceptible to immunotherapy as immunogenic tumor types [40]. Therefore, the decision whether to include immunotherapy in a treatment could be facilitated by analyzing the individual patient's immunological anti-tumor response. In this context, the sentinel node biopsy offers a unique opportunity to study early indicators of host-tumor interaction, and contributes valuable information for further conception and development of immunological treatment strategies.

## Conclusions

The axillary lymph node status is of utmost importance for breast cancer prognosis and the choice of adequate adjuvant treatment. Today, the sentinel node biopsy technique is widely used as a diagnostic tool to gain that valuable information while limiting the operative procedure to a necessary minimum. However, it is also in the sentinel node that the immunoreactivity against the tumor is initiated.

In this study, a varying extent of immunosuppression was observed, represented by a decreased expression of CD28 and CD3-ζ in certain areas of the lymph node. The concordance between both markers was highest in the areas of most pronounced down-regulation, namely the paracortical region and the germinal centers. As immunological reactivity was not distributed evenly over the different areas of the lymph node, this might illustrate the dynamic nature of the immunological response to malignant disease within the complex functional anatomy of the lymph node. Thus, the immunohistochemical analysis of the sentinel node biopsy with respect to its preserved architecture might prove an important instrument for the evaluation of interactions between the tumor and the host immune system, helping to select patients who might benefit from adjuvant immunotherapy.

## Authors' contributions

All authors participated in the study design and contributed with the collection of material and data. The microscopic analysis was carried out by AH and BG. All authors read and approved the manuscript.
